# Cost-effectiveness of a technology-supported multimodal prehabilitation program in moderate-to-high risk patients undergoing lung cancer resection: randomized controlled trial protocol

**DOI:** 10.1186/s12913-020-05078-9

**Published:** 2020-03-12

**Authors:** Anael Barberan-Garcia, Ricard Navarro-Ripoll, David Sánchez-Lorente, Jorge Moisés-Lafuente, Marc Boada, Monique Messaggi-Sartor, Laura González-Vallespí, Mar Montané-Muntané, Xavier Alsina-Restoy, Betina Campero, Manuel Lopez-Baamonde, Barbara Romano-Andrioni, Rudith Guzmán, Antonio López, Maria Jose Arguis, Josep Roca, Graciela Martinez-Palli

**Affiliations:** 1 Respiratory Medicine Department, Hospital Clínic de Barcelona, Villarroel 170, 08036 Barcelona, Catalonia Spain; 2 August Pi i Sunyer Biomedical Research Institute – IDIBAPS, University of Barcelona (UB), Barcelona, Spain; 3 Biomedical Networking Research Centre on Respiratory Diseases (CIBERES), Barcelona, Spain; 4 Anaesthesia and Intensive Care Department, Hospital Clínic de Barcelona, Barcelona, Spain; 5 Thoracic Surgery Department, Hospital Clínic de Barcelona, Barcelona, Spain; 6 Clinical Psychology Department, Hospital Clínic de Barcelona, Barcelona, Spain; 7 Endocrinology and Nutrition Department, Hospital Clínic de Barcelona, Barcelona, Spain

**Keywords:** Prehabilitation, Exercise training, Physical activity, Nutritional optimization, Smoking cessation, Cognitive behavioral therapy, Postoperative morbidity

## Abstract

**Background:**

Multimodal prehabilitation is a preoperative intervention with the objective to enhance cancer patients’ functional status which has been showed to reduce both postoperative morbidity and hospital length of stay in digestive oncologic surgery. However, in lung cancer surgery patients further studies with higher methodological quality are needed to clarify the benefits of prehabilitation. The main aim of the current protocol is to evaluate the cost-effectiveness of a multimodal prehabilitation program supported by information and communication technologies in moderate-to-high risk lung cancer patients undergoing thoracic surgery.

**Methods:**

A Quadruple Aim approach will be adopted, assessing the prehabilitation program at the following levels: i) Patients’ and professionals’ experience outcomes (by means of standardized questionnaires, focus groups and structured interviews); ii) Population health-based outcomes (e.g. hospital length of stay, number and severity of postoperative complications, peak oxygen uptake and levels of systemic inflammation); and, iii) Healthcare costs.

**Discussion:**

This study protocol should contribute not only to increase the scientific basis on prehabilitation but also to detect the main factors modulating service adoption.

**Trial registration:**

NCT04052100 (August 9, 2019).

## Background

Complete surgical resection remains the best curative option in the treatment of early stage lung cancer. However, many resectable tumors occur in patients with abnormal lung function, usually due to of the use of tobacco, having chronic obstructive pulmonary disease (COPD) and/or atherosclerotic vascular disease as underlying comorbidities. Precisely, this group of patients has an increased risk of postoperative complications and of being considered inoperable [1, 2].

Preoperative identification of patients with increased surgical risk [3] should be followed by strategies to prevent potential postoperative complications by reducing their incidence and/or severity, and thus minimizing their clinical and economic impact. Therefore, the implementation of effective preventive interventions is an important milestone to achieve due to the current economic context.

Multimodal prehabilitation is a preoperative intervention with the objective to enhance cancer patients’ functional status in order to improve clinical postoperative outcomes [4]. The main interventions included in prehabilitation programs are exercise training, nutritional optimization, psychological support and behavior change, among others. In this regard, prehabilitation has been shown to be a promising intervention to enhance aerobic capacity consequently reducing both postoperative morbidity and hospital length of stay, not only in digestive cancer patients [5–7], but also in cardiovascular surgery [8, 9]. However, in lung cancer patients undergoing thoracic surgery, further studies with larger samples and higher methodological quality are needed to clarify the potential benefits of prehabilitation [10].

It is important to highlight, that lung cancer patients candidates for thoracic surgery are more likely to benefit from prehabilitation since they usually have a significant reduction in functional capacity from multifactorial origin, namely: i) Pulmonary limitations due to COPD [11]; ii) Lean mass deficit due to the systemic effects derived from both underlying cardiopulmonary chronic conditions [12, 13] and cancer [14] and/or produced by the sedentary lifestyle presented by these patients [15]; iii) Functional alterations of cardiopulmonary comorbidities [2]; iv) Side effects of the neoadjuvant therapy (radiotherapy / chemotherapy / immunotherapy); and, v) The state of anxiety-depression associated with the diagnosis and surgery [16]. Therefore, the implementation of multimodal prehabilitation programs addressing these multifactorial etiologies in lung cancer patients undergoing thoracic surgery is of paramount importance.

The main aim of the current protocol is to evaluate the cost-effectiveness of a multimodal prehabilitation program supported by information and communication technologies (ICT) in moderate-to-high risk lung cancer patients undergoing thoracic surgery. Moreover, further secondary assessments, within a Quadruple Aim approach [17, 18], will also be performed, including: i) Patients’ experience outcomes; ii) Population health-based outcomes; iii) Costs from the hospital perspective; and, iv) Healthcare professional perspective outcomes. Additionally, an ancillary aim of the study will be to refine the ICT supporting the program in order to prepare large-scale deployment of a sustainable and modular multimodal prehabilitation services at regional (Catalonia) and European level [19].

## Methods / design

### Study design and population

**Fig. 1:**
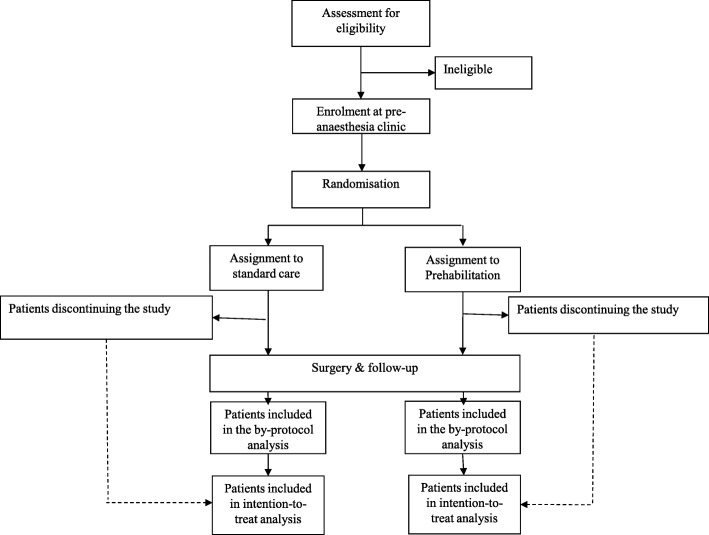
Study flow-chart

This study is a single blind randomized controlled trial. Patients will be randomized (computer-generated random numbers) on a 1:1 ratio, either to: i) a control group which will follow the standard of care established by the protocols used in our hospital; and, ii) an intervention group which, besides the standard preoperative management, will undergo a personalized multimodal prehabilitation program at Hospital Clínic de Barcelona (Catalonia) (Fig. 1). The sample of subjects will include moderate-to-high risk lung cancer patients candidates to thoracic surgery. Inclusion criteria are the following: i) Predicted postoperative forced expiratory volume in the first second and/or predicted postoperative lung diffusion capacity for carbon monoxide < 60%, and/or; ii) American Society of Anesthesiologists (ASA) classification [20] 3–4; and/or, iii) age ≥ 75 years old. Exclusion criteria are: i) Undergoing non-elective surgery; ii) known metastatic disease before surgery; iii) unstable respiratory or cardiac condition; iv) cognitive or locomotive limitations precluding adherence to the program.

The study protocol and informed consent have been evaluated and accepted by the Medical Research Ethics Committee at Hospital Clínic de Barcelona (HCB/2018/1138). The study protocol is displayed at https://clinicaltrials.gov/ct2/show/NCT04052100.

### Procedures

#### Standard care

The preoperative standard measures consist of physical activity recommendation and advice on both smoking cessation and alcohol intake reduction. Moreover, in patients presenting with anemia, the anesthesiologists will assess its etiology and treat it accordingly, and nutritional intervention will be performed by a registered dietitian in to those patients at risk of malnutrition (Malnutrition Universal Screening Tool [21] ≥2).

#### Multimodal prehabilitation program

Besides the standard preoperative management aforementioned, the intervention group will undergo a multimodal prehabilitation program. The interventions included will be patient-centered aiming to optimize patients’ preoperative health status while enhancing their empowerment and engagement. Most of the components of the program will be community-based and will be supported by a mobile solution and a technological platform for patients’ management. The main components of the program are described below.

(i) *Supervised exercise training program:* This program will consist in ambulatory exercise training sessions with two main components, namely: high-intensity endurance exercise training performed on a stationary cycle-ergometer (*Technogym® Excite Bike; Cesena; Italy*) and strength muscular training (*Technogym® Plurima Multistation Wall; Cesena; Italy*). Patients will undergo 3 sessions per week. Each endurance training session will include 5 min of warm up, 37 min of interval training, and 5 min of cool down. The interval training will combine 3 min of high-intensity pedaling and 3 min of active rest. Work-rate progress during the program will be tailored on an individual basis, according to patients’ symptoms (modified Borg scale) [22], to maximize the training effect. The strength training session will consist in 3 series of 15 repetitions for each of the following exercises: i) horizontal rowing; ii) pectoral press; and, iii) quadriceps bench. Weight progress during the program will be adapted to patients’ tolerance with the final aim of maximizing the training effect.
(ii) *Personalized program to promote physical activity:* This will be a pedometer-based program using a physical activity tracker linked to a mobile app. Main physical activity-related functionalities of the technological solution are: i) Self-monitoring of the number of steps per day and percentage of accomplishment of the objective; ii) A daily motivational message; iii) Positive reinforcement once objective of daily steps is achieved; and, iv) Exercise and physical activity educational material. International recommendations for physical activity based on the number of daily steps [23] will serve as a theoretical framework regarding the generation of goals. All the information monitored using the mobile solution will be registered in a technological platform serving as a professional backend where it will be checked and assessed by a specialized physiotherapist.
(iii) *Nutritional optimization program:* Patients will receive personalized dietary counseling from a registered dietician. Based on the initial evaluation, patients will receive recommendations of a healthy balanced diet or a diet adapted to their digestive symptoms, if present. The daily amount of protein intake capable of producing a positive nitrogen balance in these patients is estimated to be close to 2 g·Kg^− 1^·day^− 1^. This protein intake (1.5–2 g·Kg^− 1^·day^− 1^) will be ensured in patients with adequate kidney function, distributed in three main daily meals, by means of food enrichment, and/or nutritional supplementation such as whey protein powder or casein. Sufficient caloric supply will be ensured as a mean to guarantee proper protein utilization. Moreover, alcohol intake abstinence will be highly recommended and professional help offered if required. Moreover, personalized educational material as well as follow-up surveys will also be provided using the mobile app. As well as in the physical activity promotion program, the nutritional information will be registered in the technological platform and assessed by the dietician.
(iv) *Smoking cessation program:* The treatment will consist in the use of both cognitive behavioral intervention and pharmacological therapy either by varenicline and/or nicotine replacement therapies. A weekly monitoring will be performed to evaluate the evolution of their abstinence symptoms and craving control.
(v) *Cognitive behavioral therapy:* The intervention will consist on weekly group sessions conducted by a clinical health psychologist, including psychoeducation, motivational and behavioral change, self-efficacy and adherence enhancement, coping strategies acquisition and patient empowerment. The main objectives of the cognitive behavioral therapy will be to reinforce patients’ motivation, to provide coping strategies to manage stress and to foster patients’ engagement for healthy lifestyles (i.e. physical activity, nutritional habits and smoking cessation) according to the program objectives. Group sessions will be complemented by providing educational material, audio guides for coping strategies exercises and motivational text messages using the mobile solution. Patients with comorbid psychopathology will be addressed to a specialized service.

### Study variables

As stated in the introduction of the present manuscript, study outcome variables will follow a Quadruple Aim approach [17, 18]. Firstly, from the *patients’ experience perspective*, main outcome variables included will be person centeredness by the Person Centered Coordinated Experience Questionnaire [24] and continuity of care by the Nijmegen Continuity Questionnaire [25]. Moreover, focus groups and structured interviews will be also undertaken to identify facilitators and barriers to prehabilitation. Secondly, *population health-related outcome* variables will be hospital and intensive-care length of stay, number and severity of postoperative complications [26], number of hospital readmissions and emergency visits at 30 days, physical activity using Yale Physical Activity Survey (YPAS) [27], aerobic capacity measured by a standard cardiopulmonary exercise test, self-perceived health status using the Short-Form (36) health survey (SF-36) [28], anxiety and depression levels using the Hospital Anxiety and Depression score (HAD) [29], lean mass index measured by bioimpedanciometry, and levels of systemic inflammation [30] by determinations of C-reactive protein (CRP), ultrasensitive CRP, TNF-α Factor, γ-interferon and interleukins 6 and 10. Thirdly, *hospital costs* will be assessed, including: i) Patient-chargeable costs (i.e. pharmacy and blood bank); ii) Tariff-chargeable costs (i.e. medical care, diagnostic techniques, laboratories, specialist consultations, hospital length of stay and hostelry); and, iii) Other costs (i.e. support and structural costs). Last, but not least, *healthcare professionals’ perspective* will be assessed by Advancing Care coordination and Telehealth deployment at Scale (ACT@Scale) questionnaire [31]. Professionals’ perspectives (i.e. healthcare professionals, policy makers, healthcare companies’ representatives and health technology agents) will be also assessed by means of focus groups and structured interviews.

Descriptive variables will include socio-demographic, environmental, and interpersonal data, smoking status, alcohol intake, comorbidities, pulmonary function tests [32–34] and arterial blood gases. All data will be collected in a technological platform.

### Sample size calculation and statistical analysis

Sample was calculated with GRANMO program [35] taking hospital length of stay reduction as the main outcome measure. Accepting an α-risk of 0.05 and a β-risk< 0.2 in a bilateral contrast and assuming a 15% of follow-up losses and a standard deviation of 3.1, 79 patients in each group will identify a statistically significant reduction ≥1.5 days of length of hospital stay.

Data will be analyzed to explore and assess the effect of the intervention on the use of health resources and clinical impact by comparing the intervention group with the control group using hypothesis tests. The characteristics of the intervention group and the control group will be compared using Student’s t-test, Kruskal-Wallis or Chi^2^, according to the distribution of the variables. The effect of the intervention will be studied by intention to treat through regression analysis (linear, logistic, Cox or Poisson, depending on the distribution of the variable), including the exposure to the intervention as the main variable and as co-variables those in which the intervention group and the control group are different at baseline, if there were any present. A cost-effectiveness study will be carried out from the perspective of the hospital, taking into account the costs of the intervention and the expenses related to the disease during the follow-up (30 days).

## Discussion

The current manuscript reports a study protocol to assess the impact of multimodal prehabilitation in moderate-to-high risk patients undergoing lung cancer resection within a Quadruple Aim approach [17]. In this regard, the comprehensive evaluation strategy presented will provide novel information on prehabilitation at different levels, contributing to increase the scientific basis on the field, and also potentially detecting the main factors that modulated service adoption in the clinical practice.

It is well-known that novel healthcare services within an integrated care approach, such as prehabilitation in cancer surgery, are applied in complex patients and settings entailing the connectivity, alignment, and collaboration of several stakeholders and healthcare tiers. Therefore, this protocol also envisages the assessment of the practicalities for service accessibility, sustainability and scalability by means of focus groups and structured interviews with patients, caregivers and professionals.

The community setting has to be acknowledged as a cornerstone for the adoption and scalability of prehabilitation programs. The link between the hospital and the community setting should be possible thanks to ICT acting as enabling tools at three levels: monitoring, communication and patient management. In this protocol we postulate a mobile phone solution and a technological platform to optimize patients’ adherence to the work plan.

Finally, besides traditional prehabilitation outcomes (i.e. hospital length of stay, postoperative complications, cost-effectiveness), the protocol reported in this article will also explore novel aspects in the field, such as the role of systemic inflammation as a potential biomarker of prehabilitation response [30].

The approach of this study protocol should contribute to generate recommendations for transferability and refinement of prehabilitation and perioperative cancer care.

## Data Availability

Not applicable.

## Abbreviations

ACT@Scale: Advancing Care coordination and Telehealth deployment at Scale
ASA: American Society of Anesthesiologists
COPD: Chronic obstructive pulmonary disease
CRP: C-reactive protein
SF-36: Short-Form (36) health survey
HAD: Hospital Anxiety and Depression score
ICT: Information and communication technologies
YPAS: Yale Physical Activity Survey
